# From German Dental Journals

**Published:** 1896-01

**Authors:** Carl E. Klotz

**Affiliations:** St. Catharines, Ont.


ARTICLE V.

### FROM GERMAN DENTAL JOURNALS.

BY CARL E. KLOTZ, L. D. S., ST. CATHARINES, ONT.

THIOFORM.—By Dr. Edmond Prigge, dentist, Frankfort a M.—It is not to be denied that, notwithstanding the disadvantages of iodoform, we are always called back to its use again, as so far we have not found any drug that possesses all the good qualities of it without having also some of the bad. Iodoform is poisonous, and has a very disagreeable odor which is offensive to the patients. Salicylic acid, boric acid, bismuth, etc., have been tried, to replace it, but none would answer. A substitute has now been found which has none of the disagreeable qualities. It is called Thioform. The first experiments with it were made by Vet. Surgeon L. Hoffman on animals with remarkable results, which were followed by experiments by a number of physicians in their practice. I decided to try it and test its usefulness in dentistry. My first trial was with a lady patient, 35 years of age, who was troubled with empyem of the antrum, caused by the root of a first molar. When the patient came to me she complained of headache and occasionally a discharge of pus from the nose. I extracted the tooth and enlarged the opening into the antrum where there was quite a discharge of pus, and syringed the cavity with carbolized water, 1-100, till there was no more pus. I now blew into the antrum about 1 gm. of thioform powder with a chip-blower and closed the opening. The following day I found no pus in syringing the cavity with carbolized water. This treatment was kept up for several days. On the eighth day I left off syringing and left the powder in the antrum, and in twelve days had a complete cure. The second experiment was on the lower jaw of a non-commissioned officer of the regiment stationed here. He had a fistulous opening opposite the lower first molar which had been there for several years; it had now broken out through the cheek. I extracted the offending tooth, and carefully removed the degenerated parts around the cheek fistula, made two incisions according to Jane's method and put in two stitches, treated with thioform powder and put on a bandage. After two days I removed the stitches and in eight days had a complete cure. My next trial was on pulp tissue. For this purpose I made a paste of

| | |
|---|---|
| Thioform . . . . . . . . | 2 o. |
| Glycerine . . . . . . . . | q. s. f. p. Mollis. |

With this I filled the root canal of a lower second bicuspid of a young lady, after I had carefully removed the pulp and covered it with a temporary filling of gutta percha. After seven weeks I removed the temporary filling and could not detect the slightest trace of a disagreeable odor, nor had the patient experienced any pain. The canal was now filled with chl. zinc cement and the cavity with a gold filling. With this same patient I had a still more venturesome trial, which has so far also proved successful. I amputated the pulp of an upper second molar according to the Herbst method, but before I covered it with tin foil I placed a little thioform paste into the pulp chamber, covered it with tin foil and filled the cavity. In this nanner I have treated a number of patients since, and am well satisfied with the results. The principal advantage of the thioform is that it is not corrosive or poisonous; it is a hæmostatic and slightly anæsthetic. In other respects it has all the good qualities of iodoform.

A CASE IN PRACTICE.—Removal of a supernumerary canine by Ph. Zundel. Patient, a strong young man of 22 years of age, a baker by trade, came to the office with the left side of his face badly swollen. Two years previous he had the left central and lateral extracted and replaced by artificial teeth on a vulcanite plate. Excepting the loss of the teeth there was no further irregularity to be seen. According to the patient's statement, he has had this swollen face more or less since he had the artificial teeth inserted. My first thought was that there might be part of either tooth left in the alveolus, causing the inflammation, but this was not the case, for upon closer examination I found marked fluctuations of the tissues. I lanced the inflamed part, and out of it flowed a greenish liquid that had an odor somewhat similar to peppermint. After the wound was thoroughly disinfected and probed, I found a smooth bony substance in a horizontal position, about 1½ cm. in length. In forcing an elevator into the opening a supernumerary canine was removed. The wound was treated with antiseptics, and healed in fourteen days.—*Monatsschrift Deutscher Zahu Kunstler.*

NERVOUS ODONTALGIA.—Dr. Gretner has used of late in a number of cases of violent toothache the following remedy with very good success:

R Antefebrin . . . 0.2C
Phenacetini . . . 0.5

M. f. pulv., D. t. dis No. 6. S. Three times daily, 1 powder.

Particularly satisfactory were the effects of the powder with a patient in the second month of pregnancy. As is known, toothache at this period is remarkable for its stubborness. She had suffered excruciating pain for three days and three sleepless nights. After taking the first powder she had relief and slept all night, and after the second powder the following day, the pain had entirely left, and no recurrence since.

PORCELAIN AMALGAM CROWN.—Dr. Nitus Nuki, of Temesvar, Hungary, says: A very simple bicuspid crown is made as follows—Prepare the root same as for any crown, take an impression, select a plate tooth and grind to fit the model. Prepare amalgam same as for filling a tooth, but rather more plastic, pack between and around pins and pivot, building up the inner cusp; when hard, polish and cement to place.

NERVE PASTE.—Dr. Diack considers morphia in a nerve paste ineffective, likewise tannin, aristol, iodoform, cocain or oil of cloves. He adds belladonna ext. to the arsenious acid, two parts of the former to one of the latter, and mixes them into a paste with glycerin. Belladonna acts on the sensitive nerves, and no pain follows the application of the paste.

FORMULA FOR ODONTOL.—

R. Cocain Hydrochloric,
Aq. Laurocer,
Tinct. Arnica . . . . . . aa. 1.00
Liq. Ammon. Acetic . . . 10.00

Apply on a pellet of cotton in the cavity of the tooth.

ANOTHER TOOTHACHE REMEDY.—

Acid Carbol. Crystals,

Cocain Mur,

Menthol . . . . . . . . . aa. 1.00

Glycerine . . . . . . . . 20.00

—*Zahuarstliches Wochenblatt.—Dominion Dental Journal.*

